# From Meaning, Spirituality, and Religion in Acute Psychiatry to Public Health: A ‘Dual Motor’ Model

**DOI:** 10.3390/ijerph23020176

**Published:** 2026-01-30

**Authors:** Bart van den Brink, Linda van Parijs, Joke C. van Nieuw Amerongen-Meeuse, Janieke I. Tjepkema, Rogier Hoenders

**Affiliations:** 1 GGz Centraal, Mental Health Care, 3818 EW Amersfoort, The Netherlands; 2 Department of Practical Theology, Theological University Apeldoorn, 7316 BT Apeldoorn, The Netherlands; 3 University Medical Central Utrecht, 3584 CX Utrecht, The Netherlands; i.j.tjepkema@students.uu.nl; 4 Altrecht Mental Health Care, 3512 PG Utrecht, The Netherlands; 5 Faculty of Social Sciences and Humanities, Lived Religion and Society, Vrije Universiteit Amsterdam, 1081 HV Amsterdam, The Netherlands; 6 Center for Research and Innovation in Christian Mental Health Care, 3871 MR Hoevelaken, The Netherlands; 7 Lentis Mental Health Care, 9725 AG Groningen, The Netherlands; h.j.r.hoenders@rug.nl; 8 Faculty of Religion, Culture and Society, University of Groningen, 9712 CP Groningen, The Netherlands

**Keywords:** spiritually integrated therapy, SPIRIT, cognitive behavioral therapy, aftercare, existential recovery

## Abstract

**Highlights:**

**Public health relevance—how does this work relate to a public health issue?**

- Meaning, spirituality, and religion are related to mental health and recovery from mental illness.
- Group-based therapies that integrate meaning, spirituality, and religion, such as SPIRIT, can contribute to recovery from mental illness and increase wellbeing and mental health at the population level.

**Public health significance—why is this work of significance to public health?**

- This study provides insight into the perceived effects of spiritually integrated cognitive behavioral therapy and identifies factors needed to sustain these effects over time.
- By examining needs in terms of aftercare, this research addresses a knowledge gap on how psychotherapeutic interventions can contribute sustainably to (existential) recovery.

**Public health implications—what are the key implications for practice, policy, and research?**

- A ‘dual-motor’ model is proposed: meaning-focused therapies promote both coping and a broader recovery-oriented contextualization in therapy and at home.
- The integration of meaning, spirituality, and religion in acute psychiatry requires team-wide implementation.
- Structural attention to follow-up and integration into subsequent treatment may contribute to more sustainable recovery.

**Abstract:**

Spiritually integrated group therapy aims to support coping, meaning-making, and existential recovery in patients receiving psychiatric care. SPIRIT (Spiritual Psychotherapy for Inpatient, Residential and Intensive Treatment) is a structured, flexible protocol implementing this approach into intensive or residential settings. This study examines (1) the impact of SPIRIT on patients’ lives and (2) their needs in terms of aftercare to determine whether and how its benefits can be sustained long term. Data were collected from multiple sources: patient evaluation forms (n = 118); in-depth interviews with patients (n = 19) and caregivers providing the therapy (n = 8); and two focus groups with both caregivers and patients. Transcripts were analyzed using qualitative content analysis. Results indicate that for most participants, the therapy positively impacted their lives through increased awareness or behavioral change, highlighting the relevance of maintaining these insights after group therapy, either at home or within the treatment setting. We recommend broader training of mental health professionals, and the introduction of programs like these to the entire care team to ensure awareness and support. A ‘dual motor model’ is proposed. Addressing religious, spiritual, and meaning-related themes in ongoing therapy and the psychosocial and pastoral support network can support recovery both by reducing symptoms and by fostering a health-promoting context.

## 1. Introduction

According to recent data from the World Health Organization (WHO), over one billion people worldwide live with mental health disorders, with conditions such as anxiety and depression exacting a heavy human and economic toll [1,2]. In recent decades, a more recovery-oriented approach encompassing self-actualization and connected living eclipsed a previously more symptom-oriented and professionally guided approach. Therapies integrating meaning, spirituality and religiosity (MSR) are increasingly recognized as a valuable part of a recovery-oriented approach in mental health care, as they address patients’ needs for MSR, and coping with illness [3,4]. Examples include religiously integrated cognitive behavioral therapy [5], spiritually augmented acceptance and commitment therapy [6], and mindfulness-based interventions [7]. These approaches have been well studied in English-speaking countries, particularly the United States, where evidence suggests they can improve coping, psychological functioning, and patients’ sense of meaning [8].

In European countries, however, engagement with MSR-integrated therapies has been more limited. Fewer studies have been conducted, and in secular contexts such as the Netherlands, there are relatively few tools available. Nevertheless, there is evidence that patients in these settings also express a need for interventions addressing MSR [9]. Most established therapies are designed for outpatient care, yet there is increasing demand for support in acute and inpatient settings.

SPIRIT (Spiritual Psychotherapy for Inpatient, Residential, and Intensive Treatment) is a unique, spiritually integrated open group therapy based on principles of cognitive behavioral therapy (CBT) and developed for acute psychiatric settings, that proved feasible and clinically relevant in the United States [8]. SPIRIT-CBT is theoretically grounded in spiritually integrated psychotherapy, recovery-oriented public mental health care, and transdiagnostic evidence-based approaches, mainly cognitive-behavioral and dialectical behavior therapy. It aims to treat disorders and promote health. The program was developed to meet an unmet need in acute psychiatric settings by providing a structured yet flexible, clinician-led group intervention that addresses existential dimensions of recovery across diagnostic categories [10]. Initial evaluations indicate that patients experience the program as supportive and meaningful [11]. SPIRIT-CBT supports recovery by helping patients identify MSR resources and recognize spiritual struggles that contribute to psychological distress, across diagnostic categories and levels of clinical intensity.

In the Netherlands, religious and/or spiritual care needs have been clearly demonstrated, indicating a need for interventions that address existential and meaning-related themes [9]. A literal Dutch translation of the SPIRIT protocol was well received and led to the development of a culturally adapted version with ten handouts incorporating meaning and a recovery-oriented approach [12]. Previous studies have primarily focused on short-term outcomes using quantitative or qualitative methods [12,13,14].

From the perspectives of public mental health, recovery-oriented care, spiritually integrated care, and continuity of psychiatric care, previous research has highlighted the importance of interventions that address both individual outcomes and service-level considerations [2,3,4,15,16,17]. However, since MSR-integrated therapy for acute and intensive psychiatry is a new approach, its impact on clients’ needs exploration, as does the potential need for follow-up care and how this should be provided. Thus far no studies have had a focus on aftercare for any MSR-integrated interventions to our knowledge. SPIRIT-CBT offers the opportunity to study the impact and aftercare needs of an MSR-integrated intervention. This creates a point of focus to integrate these often separately approached academic discourses from a real-life perspective. It also offers a possibility to explore the potential of this sort of psychotherapeutic intervention to benefit not only the individual but also mental health services, recovery, and well-being at the level of populations or service systems.

Given the importance of meaning and existential recovery for mental health, it is crucial to examine whether MSR-integrated therapy produces lasting impact, thereby supporting continuity of care and preventing relapse. This study therefore examines (1) the impact of SPIRIT-CBT on patients’ lives and (2) their needs for aftercare, in order to determine how these insights can inform the delivery of mental health services, foster recovery, and enhance well-being at the level of populations and service systems. Specifically, this study investigates: (a) whether participation in SPIRIT group CBT is experienced as supportive for recovery; (b) whether MSR, both in private activities and networks as well as in therapy, is effectively integrated and contributes to coping and the experience of meaning; and (c) whether SPIRIT-CBT influences broader determinants of health, such as psychological, social, and environmental factors, thereby fostering the long-term development of positive coping and a supportive environment.

## 2. Materials and Methods

This study was conducted in The Netherlands as part of a collaborative project between the Center for Research and Innovation in Christian Mental Health Care (Kicg) and four mental health care institutions in The Netherlands: GGz Centraal, Altrecht Mental Health Care, Eleos and De Hoop. The study was conducted in accordance with the Declaration of Helsinki and was considered compliant by the Medical Ethical Committee of the University Medical Center Utrecht (protocol code NR 22-1041/DB; 5 January 2023). The study was approved by the ethical committees of the participating organizations. The project was funded by ZonMw (research number 10960102310035), a Dutch national organization for health research and innovation.

### 2.1. Intervention

Spiritual Psychotherapy for Inpatient, Residential and Intensive Treatment (SPIRIT) is an open thematic CBT group psychotherapy that was delivered weekly or biweekly across four Dutch intensive mental health care settings as part of the treatment program: Altrecht (open inpatient unit), GGz Centraal (acute day program), de Hoop (inpatient substance use treatment), and Eleos (open inpatient unit). Groups were usually led by two clinicians of different disciplines—psychiatrists, nurses, social workers, or psychologists. During each session, the central question was ‘How do meaning and MSR affect my mental health, and how do my mental health challenges affect my outlook on life?’ Patients were encouraged to consider the relationship between the two and to reflect on it. Then, for the second part of the session, one of ten handouts was selected to provide patients with practical tools to apply beyond the session. These handouts covered topics like coping and reframing, spiritual or existential struggles, or autonomy and responsibility. At Altrecht, participation in SPIRIT-groups was optional, whereas at the other sites it was scheduled as part of routine care; participation has never been mandatory. Each group typically included three to ten participants. In some settings, patients attended a single session, while in others they participated in multiple sessions.

### 2.2. Study Design

Participants were all acute mental health patients with various diagnoses, receiving either clinical care or day-care. We employed a qualitative research design, making use of data triangulation to enhance the validity and depth of the findings [18]. Three complementary data sources were included: (1) In-depth interviews with patients (n = 19) and patient evaluation forms (n = 118); (2) in-depth interviews with mental health professionals (n = 8); (3) two focus groups with a diverse group of stakeholders, including patients, group leaders, and researchers.

### 2.3. Participants and Recruitment

All patients attending SPIRIT-CBT group sessions were invited to complete an anonymous evaluation form at the end of a group session, consisting of five questions. No personal identifiers, such as gender, age, or diagnosis, were collected. The form also asked whether participants could be contacted for a follow-up interview. Each participant completed a maximum of one form, which referred to the most recent session they had attended. The response rate for the evaluation forms was approximately 50%, and around 30% of respondents indicated willingness to participate in an interview. Because of the low threshold for filling out an evaluation form, this is a close estimation only. All individuals who agreed to be contacted subsequently provided verbal consent during a follow-up phone call. In total, three participants from Eleos, three from de Hoop, four from GGz Centraal, and nine from Altrecht were interviewed. For the analysis, contributions from the four institutions were weighted equally to account for the slightly higher representation from one site. Additionally, eight group leaders were invited to participate in an interview, and all agreed to do so. For the focus groups, a selection of group leaders, patients, and researchers was approached, and two dates were set for those who were available to participate in the online sessions.

### 2.4. Data Collection

The evaluation forms contained five open-ended questions. The open-ended items focused on patients’ experiences with the intervention, perceived benefits or possible struggles, and suggestions for improvement. Semi-structured interview guides were developed, addressing experiences with the intervention, perceived impact, barriers and facilitators, and recommendations for further development. Attention was paid to the way in which elements of the group therapy were applied after the intensive treatment patients received, in the broader context of their lives. Interviews were conducted individually, either face-to-face or online, and lasted approximately 45–60 min. All interviews were audio-recorded and transcribed verbatim; automatic transcripts were carefully checked and corrected manually. Two online focus groups were organized, each consisting of a mix of patients, group leaders, and researchers. The discussions focused on shared experiences with the intervention, perceived added value, and ideas for further implementation in practice. Each focus group lasted about 60 min and was facilitated by an experienced moderator.

### 2.5. Data Analysis

All qualitative data (interview transcripts, focus group transcripts, and open-ended evaluation form responses) were analyzed using content analysis [19]. Because data were collected using different methods, each type of data was analyzed in a way that best suited its format and content. Evaluation forms (n = 118) provided a brief, anonymous overview of patients’ perceived benefits. Interviews (n = 19) allowed in-depth exploration of experiences from both patient and clinician perspectives. Focus groups (n = 2) provided the opportunity to discuss issues collectively and to triangulate findings across participants. Interview data were analyzed using ATLAS.ti version 25 (ATLAS.ti Scientific Software Development GmbH, Berlin, Germany). The analysis proceeded in several iterative phases: open coding, in which meaningful segments were coded inductively; axial coding, in which codes were clustered into broader categories and themes; and selective coding, in which a set of core codes was refined in relation to the research question. Consistent with a team-based approach to qualitative analysis [20], the first, second, and third authors collaboratively conducted the analysis. They compared coding decisions, discussed interpretations, and refined categories. Analytic memos were used throughout to capture reflections and emerging insights. Discrepancies were resolved through discussion until consensus was reached, thereby enhancing the credibility and trustworthiness of the findings [21]. This systematic and collaborative approach ensured that the analysis was both rigorous and grounded in the data, while also incorporating multiple perspectives from the research team.

## 3. Results

Results were divided into the topics: participant characteristics, how SPIRIT-CBT has an impact on patient’s lives; why follow-up after the therapy might be relevant; how patients perform this in private context; and how this could take place in treatment context. Codes and subthemes were summarized in Table 1.

### 3.1. Participants

A total of 118 participants filled in the evaluation forms, but as this was low-threshold survey, no data about sex, diagnoses, etc., were collected. Of the evaluation forms, 85% of the participants were mainly positive and the others neutral. Of the patient interviews, 18 out of 19 were positive about the intervention. The other participant considered group therapy too intensive and too much and withdrew from the group before it ended. The characteristics of the interviewed clients and caregivers are summarized in Table 2. During the focus groups no demographics were collected.

### 3.2. Impact of the Intervention

Most participants reported that attending SPIRIT-CBT groups had influenced them in some way. The therapy appeared to impact patients in various domains: by touching them on a personal level, influencing how they cope with illness and distress, contributing to a meaningful change, and by shaping the importance of meaning, spirituality and/or religiosity (MSR) in their lives.

Touching on a personal level: “Just from day to day. Enjoying the little things, like being with my parents.” (Patient interview).

Influencing coping: “My husband and I now give each other a compliment a day.” (Patient evaluation form).

Contributing to meaningful change: “I think I’m more aware that I’m not the little bird that can be crushed at any moment. I have wings, so I can fly away.” (Patient interview).

“It opened a little door again. It gave me something to reflect on.” (Patient interview)

Shaping the importance of MSR in their lives: “NN [group leader] looked with me at a site where you can see exactly which church holds which beliefs, and so on.” (Patient interview).

SPIRIT-CBT contributed to greater awareness of personal meaning, spirituality, and worldview. For some participants, this led to subtle shifts in attitude or interest—such as increased openness, renewed engagement, or a sense of gentleness. Others primarily experienced recognition and affirmation of what was already present.

### 3.3. Why Follow-Up?

Several patient participants mentioned the importance of aftercare (either in the context of treatment or in the context of their social environment) when they followed SPIRIT group therapy, for example, to obtain insights in how to proceed as an individual.

“I thought, like, what if I don’t come back here and have to go on without therapy—how on earth am I going to manage that? […] And then, when that ends—which is, of course, completely normal and actually fine—you’re left thinking: okay… now what?” (Patient interview)

Other patients, participating in a focus group pointed out the barriers to addressing the MSR themes, including the taboo surrounding some topics as a reason for the need of aftercare. They emphasized that the topics do not emerge spontaneously and that the group session was not long enough to cover all the themes, not all issues could be discussed: “Yes, after SPIRIT, you kind of fall back into everyday society, and that sense of wonder and broadening kind of fades away again because there’s less support available in that area. And I think that also ties in with the fact that spiritual, meaningful, and religious matters carry a huge stigma, especially when combined with mental health issues. I believe it’s still a taboo subject. […] It feels like a big step to even bring it up in therapy, for example, especially if your therapist has no connection with it or doesn’t know what questions to ask. Then it feels like you’re back to square one. You end up nostalgically thinking back to the SPIRIT group—but then you wonder, well, what now?” (Patient in focus group).

“There were definitely more topics that touched me as well, but it’s so strange that we never talk about these things. About what illness or life experiences do to you. It’s something everyone goes through in some way, but we just never talk about it.” (Patient interview)

In addition, caregivers emphasized the relevance of aftercare in the context of treatment. They illustrated that SPIRIT-CBT often functioned as a starting point and that it would be necessary to go on with the themes addressed and finetune this in individual contexts: “Things really do get touched upon, and I genuinely think that’s when a professional is needed—someone who can think on a more overarching level, to help distill the underlying questions or needs, and to provide direction or suggest possible options. Otherwise, I think some important things do get stirred up, but then they just… get buried again, so to speak. And that would be a real shame.” (Caregiver interview 1).

### 3.4. Follow-Up in Private Context

Since the need to proceed with themes addressed during SPIRIT therapy was clearly present among the patient research population, several participants mentioned practicing continuing with SPIRIT-CBT after the group sessions by filling out extra hand-outs or writing down and reminding themselves of helpful texts: “With some of the themes, we’d also ask things like, ‘Hey, what inspiring text are you bringing with you this week?’ Or, “What kind of meditation are you planning to use this week?’ I thought that was a really nice addition… and it really fit well with the rest of the week, because it allowed you to actually put things into practice.”

Others showed that they used insights with their relatives, discussing the topics with their partner, children or other family members. A different wish of patients was to continue with the group in a social or faith community, though they did not know how to organize this: “I’m sad that the group is ending. It would be wonderful if something similar existed outside of mental health care, for example in a religious or informal setting.”

However, most of these participants would highly appreciate it if follow-up possibilities would also be present in treatment context.

### 3.5. Follow-Up in Treatment Context

Taken together, while patients appreciated the opportunity to apply the insights derived from SPIRIT-CBT in their daily lives and treatment, the combination of barriers—such as caregivers’ limited awareness or preparedness to discuss MSR topics and the experienced taboo—suggests that many patients would value caregivers taking the initiative to actively address MSR topics in aftercare.

The practice and preferences regarding SPIRIT-CBT follow-up in the treatment context can be organized stepwise. First, several inpatients or day-care patients who preferred follow-up spoke afterwards with one of the group leaders. Sometimes this was to further process insights from the group, and sometimes to explore how these insights could be connected to their personal context: “That I can talk things over with the nursing staff afterwards really helps.” (Patient, interview).

Second, several patients chose to follow up with a spiritual counselor, or were advised to do so. This was also the case when the group setting did not suit them well: “And the spiritual counselor—there was a good connection, so to speak […] I also advised that he should have an individual conversation with them.”

Third, many patients and caregivers mentioned that it might be relevant to integrate MSR elements into further treatment and to integrate them in their treatment goals and conversations with their personal caregiver: “I could work with my caregiver on certain personal goals and link them to meaning—like, how can I work on my goals through a sense of meaning? Because looking back now, I really missed that connection.” (Patient in focus group).

However, they also noted that this remains challenging and requires careful consideration of how best to implement it. In addition, patients had ideas how to perform this: “I also think it could help to have something—a course or a handout—for professionals who don’t necessarily lead the group but do work with people who are interested in these kinds of themes. At the very least, it would give professionals a bit of support, showing them that it’s not dangerous to have a conversation about this with someone. I think that could be helpful.” (Patient, focus group).

Overall, the findings illustrate that SPIRIT-CBT has a meaningful impact on patients’ lives, while also highlighting a clear need for structured follow-up—both in private and treatment contexts—to consolidate insights, support coping, and integrate spirituality and meaning into ongoing care.

## 4. Discussion

Patients who participate in SPIRIT-CBT group therapy generally value this experience, as shown by previous research in the United States [11] and the Netherlands [12]. In-depth analyses of why patients find the therapy helpful are still ongoing. The current study underscores that patients benefit not only from the content of the sessions, but also from the shared experiences, normalization of struggles, and peer support that group therapy provides [22]. These elements foster a sense of community and existential understanding that is unique to group treatment compared with individual therapy. This is bolstered by the MSR themes in the handouts, like, for example connectedness and MSR-based answers to suffering or grief. Patients mentioned essential ways in which participating in this MSR-based intervention had impacted their lives. The participants’ reports of increased awareness and new routines reflect how meaning making serves as an active coping resource in chronic illness, supporting adaptive responses rather than passive acceptance [23]. The opening of a ‘little door’ to reflection mirrors findings that making sense of illness through spiritual lenses can facilitate coping transitions. Carefully evaluating aftercare needs ensures continuity of care and prevents negative outcomes after discharge.

SPIRIT-CBT is not only a psychotherapeutic intervention with individual benefits, but an intervention that might potentially affect the way mental health care is viewed and shaped. Being a health-promoting, recovery-oriented program, the intervention helps clients to redefine one’s relationship with the disease, oneself, significant others, and perhaps higher powers. By creating attention to themes beyond symptoms from the very beginning, the therapy fosters both the therapeutic relationship and broader integration into the care context, suggesting that early attention to these themes in intake discussions and care planning, combined with timely engagement of community support, may enhance recovery-oriented practice. In this way, it also impacts the environment of the participants. The therapy helps to uncover struggles, find and develop supportive insights to deal with disease, but also challenges behavior connected to the disease and core beliefs fueling this behavior. This is what makes SPIRIT-CBT a recovery-oriented therapeutic intervention.

The results of the current study underscore the importance of ensuring that the insights gained during the program are not lost after its completion but are meaningfully integrated into follow-up care and daily life. Insights from the identified aftercare needs can guide continuity of care by informing follow-up plans, support discharge planning by specifying the interventions and contacts patients need after leaving intensive care. In addition, they can shape community-based support by highlighting which coping and meaning-making strategies should be reinforced in patients’ networks. This may help prevent deterioration or relapse but also support long-term recovery and social participation [24].

An advantage of SPIRIT-CBT is that it is applicable for patients with all types of diagnoses and life perspectives. The therapy encourages a broader view on recovery that incorporates MSR dimensions. At the same time, raising awareness of individual needs and struggles may make disparities in available support more salient, potentially leading to temporary increases in distress or symptoms. This underscores the importance of considering how access to interventions and follow-up care within routine public services may contribute to differential recovery trajectories and health inequalities. The current study indicates that, for at least some patients, integrating these insights and themes into personal treatment may require individual attention within mental health care. This is consistent with research stating that linking group therapy with individual treatment goals is essential for fostering recovery [25]. Research shows that post-intensive psychiatric treatment support is crucial for sustaining recovery and integrating therapeutic insights [26].

The finding that patients may prefer aftercare following SPIRIT-CBT can be understood within this broader context and aligns with the increasingly prevalent person-centered approach in mental health care [15,22]. When individuals receive multiple forms of treatment, it becomes particularly important that these are well integrated [25]. Group therapy has intrinsic value—providing peer support, normalization, and shared experiences [22]—and should not be replaced; rather, the connection between group therapy and individual treatment is essential. In the Netherlands, spiritual care has often been provided as an open space without structured reporting or linkage to treatment. Though this is beneficial for some, this lack of connection has been experienced as a problem by others [16]. Broadening the scope, a holistic approach and adequate aftercare in any form can promote long-term well-being and recovery. Every illness carries a tendency for self-focus, and MSR can help broaden the patient’s perspective, allowing symptoms to be acknowledged and integrated into a wider context. Even through this indirect route, which does not target symptoms directly, clinical, symptomatic, and social recovery can be expected, potentially reducing hospitalizations, improving quality of life, and enabling more effective engagement with community-based services and groups. This is achieved not so much by appealing to knowledge of the illness itself (although some psychoeducation on the relationship between MSR and symptoms is included in the module), but by fostering understanding of coping with suffering, vulnerability, and brokenness, and by sharing this knowledge with significant others and caregivers. The current study therefore contributes by offering guidance on bridging this gap between spiritual care and formal treatment.

The current study provides concrete suggestions for further advancing the integration of MSR in mental health care. The use of qualitative data allowed for an in-depth exploration of participants’ experiences, and the threefold method of data collection ensured a broad spectrum of perspectives. However, the qualitative approach also limited the number of participants, and it is likely that the most motivated patients were more willing to participate, introducing potential selection bias. Therefore, the expressed preferences may not represent all participants, though they remain relevant and warrant attention. Although the present study is based on data from the Dutch context, previous work has shown that such protocols can be successfully translated and culturally adapted [12]. Its flexible design suggests that the intervention and its approach to aftercare could also be applied in other European countries or globally, if adaptations are made to meet the specific needs of patients in different cultural and service contexts. Some aftercare needs, such as follow-up support, coping strategies, and meaning-making, are likely to be broadly applicable across countries, whereas others—such as engagement with specific community resources or family systems—may vary according to local cultural and healthcare contexts. This highlights both the potential for international applicability and the importance of context-sensitive adaptation.

Based on our findings, we offer several public health-oriented recommendations. First, mental health services may consider systematically incorporating MSR-integrated approaches and tailored MSR-integrated aftercare plans to support long-term recovery and prevent relapse. Second, early engagement with patients’ social networks and community resources can enhance continuity of care and recovery-oriented outcomes. Third, future research might examine implementation strategies, cost-effectiveness, and cultural adaptation of such interventions to guide policymakers, service managers, and practitioners in improving population-level mental health outcomes. Fourth, we recommend that institutions offering SPIRIT-CBT provide informational presentations to the entire staff of the units where it is implemented. When more clinicians are trained to provide the therapy, this may also add to broad sustainability. Fifth, group leaders may dedicate a few minutes of each session to discuss how patients wish to proceed after the group, while in fixed groups, a final session could focus entirely on this topic. This ensures that insights gained in the group are integrated into individual treatment plans and goals [17] and helps patients address these themes in individual care when necessary [26].

The impact and aftercare perspectives of SPIRIT-CBT, as explored in this study, demonstrate the need for a ‘dual motor’ model of MSR-informed interventions. MSR-informed interventions can (1) bring about recovery at the symptom level, and (2) bring about recovery by providing a ‘harbor’ transcending the symptom level. MSR-interventions do not only help clients to deal and cope with their symptoms in a more adaptive way, but also in regaining a sense of meaning and purpose in life [27]. Core MSR themes (e.g., trust, connectedness, faith, forgiveness) support recovery from symptoms, and, perhaps even more importantly, enhance self-efficacy, reconnection to significant others, and the restoration of a supportive network [28]. Clients who find out that health and physical activity are important for them can start participating in a weekly sportive activity. Clients who want to reconnect with religious sources of meaning can start daily meditation or visit a worship service. Even for those who are not able to attain recovery at the symptom level, MSR can provide a framework of meaning and an MSR-driven community network that helps to ‘harbor’ them and their problems. This ‘dual motor’ model is closely related to the ‘salutogenesis’ concept as conceptualized by Anonovsky, versus the ‘pathogenesis’ view. This concept stresses also the importance of focusing on people’s resources and dimensions to create health and well-being instead of a focus on risks, ill health, and disease [29]. SPIRIT-CBT could play a role in both aspects.

Future research could examine the benefits of SPIRIT-CBT group therapy with and without aftercare, to better understand the added value of follow-up support. Furthermore, documenting the experiences of both patients and group leaders could provide valuable insights into the therapy process. Quantitative studies may explore outcomes such as MSR coping [30], existential recovery [31], MSR-related care needs [9], and MSR-related struggles [32]. In addition, replication of this research in other countries could help determine how SPIRIT-CBT can be adapted for different target populations. Together, these steps can enhance a ‘dual motor’ contribution to recovery and help to find a more adequate answer to bring mental health out of its status as a ‘public health crisis’ [33].

## 5. Conclusions

SPIRIT-CBT group therapy is a psychotherapeutic intervention, with both health-promoting and recovery-oriented impacts. The therapy has a meaningful impact on patients’ lives, enhancing personal awareness, coping strategies, and engagement with meaning, spirituality and religion. The current research indicates the potential of MSR-integrated therapy to foster recovery and prevent deterioration after therapy ends. Participants highlighted a clear need for follow-up, both in private and treatment contexts, to consolidate insights and integrate them into daily life and ongoing care. SPIRIT-CBT, and other MSR-integrated care, can alter connections to close contacts and help (re)start an MSR-driven community network or activity, strengthening support from the psychosocial network. We propose a ‘dual-motor’ model for the effect of MSR-focused therapies, as they potentially have a supportive influence both at the level of symptoms and at the level of stimulating a broader recovery-oriented context in therapy and at home. Implementing structured aftercare, training more clinicians, and connecting group therapy with individual treatment plans can enhance the therapy’s contribution to existential recovery. Future research may examine the added value of follow-up support and explore adaptation for different patient populations.

## Figures and Tables

**Table 1:** Codes and subcodes concerning SPIRIT-CBT impact and aftercare.

Main Theme	Subtheme
Impact of the intervention	Touching on a personal level
	Helping to cope with illness and complaints
	Contributing to a meaningful change
	Reshaping importance of meaning, spirituality and/or religion (MSR)
Relevance of Follow-Up	Need for guidance after SPIRIT group
	Taboo of spiritual and meaning-making topics
Follow-Up in Private Context	Self-help and personal exercises
	Discussion in social or faith community
Individual follow-Up in treatment	Conversations with group leaders or nursing staff
	Spiritual counselor/individual follow-up
	Integration into treatment goals
	Individual conversations with personal caregiver

**Table 2:** Participant characteristics of the interviews.

	Clients (n = 19)	Caregivers (n = 8)
Sex (n male/female/nonbinary)	8/10/1	3/5/0
Age (mean, SD)	41 (14)	37 (12)
Context group		
Clinical unit	16	5
Day care	3	3
Outlook on life		
Christian, orthodox	4	1
Christian, evangelical	2	3
Christian, liberal	-	2
Islamic	1	-
Nonreligious	12	2
Diagnoses (patients, self-reported)		
Depression	11	
PTSD	8	
Substance use	3	
Anxiety disorder	2	
Autism	2	
ADHD	1	
Anorexia	2	
Psychotic disorder	3	
Bipolar disorder	1	
Occupation (caregivers)		
Psychiatrist (/trainee)		2
Psychologist (/trainee)		2
Social worker		3
Nurse		1

## Data Availability

Data is anonymized and stored at the Center for Research and Innovation of Christian Mental Health care and available at reasonable request.

## Abbreviations

The following abbreviations are used in this manuscript:

SPIRIT	Spiritual Psychotherapy for Inpatient, Residential, and Intensive Treatment
CBT	Cognitive Behavioral Therapy
MSR	Meaning, Spirituality, and Religion
UMCU	University Medical Center Utrecht
WMO	Wet Medisch-Wetenschappelijk Onderzoek met mensen (Dutch Medical Research Involving Human Subjects Act)
KICG	Center for Research and Innovation in Christian Mental Health Care
GGz	Geestelijke Gezondheidszorg (Dutch for Mental Health Care)

## References

[1] World Health Organization (2024). World Mental Health Today.

[2] World Health Organization (2024). Mental Health Atlas 2024.

[3] Bouwhuis-Van Keulen A.J., Koelen J., Eurelings-Bontekoe L., Hoekstra-Oomen C., Glas G. (2024). The evaluation of religious and spirituality-based therapy compared to standard treatment in mental health care: A multi-level meta-analysis of randomized controlled trials. Psychother. Res..

[4] Captari L.E., Hook J.N., Hoyt W., Davis D.E., McElroy-Heltzel S.E., Worthington E.L. (2018). Integrating clients’ religion and spirituality within psychotherapy: A comprehensive meta-analysis. J. Clin. Psychol..

[5] Pearce M.J., Koenig H.G., Robins C.J., Nelson B., Shaw S.F., Cohen H.J., King M.B. (2015). Religiously integrated cognitive behavioral therapy: A new method of treatment for major depression in patients with chronic medical illness. Psychotherapy.

[6] Wade N.G., Worthington E.L., Vogel D.L. (2007). Effectiveness of religiously tailored interventions in clinical settings: A meta-analysis. Psychother. Res..

[7] Oman D., Driskill J.D., Shonin E., Van Gordon W., Griffiths M.D. (2015). Mindfulness in Christian and Buddhist spiritual traditions: Connections with mental and physical health. Mindfulness and Buddhist-Derived Approaches in Mental Health and Addiction.

[8] Currier J.M., Bounds E.M., Matsuo H., vanOyen Witvliet C., Abernethy A.D., VanHarn K., Schnitker S.A. (2024). Temporal associations between meaning in life, ultimate meaning struggles, and mental health outcomes in a spiritually integrated inpatient program. J. Clin. Psychol..

[9] van Nieuw Amerongen-Meeuse J.C., Schaap-Jonker H., Westerbroek G., Anbeek C., Braam A.W. (2020). Conversations and beyond: Religious/Spiritual care needs among clinical mental health patients in the Netherlands. J. Nerv. Ment. Dis..

[10] Rosmarin D.H., Salcone S., Harper D., Forester B.P. (2019). Spiritual Psychotherapy for Inpatient, Residential, and Intensive Treatment. Am. J. Psychother..

[11] Rosmarin D.H., Salcone S., Harper D.G., Forester B. (2021). Predictors of patients’ responses to Spiritual Psychotherapy for Inpatient, Residential, and Intensive Treatment (SPIRIT). Psychiatr. Serv..

[12] Van Nieuw Amerongen-Meeuse J.C., Ouwehand E., Graaf N.D., Parijs L.V., Schaap-Jonker H., Braam A., Verhagen P.J., Rosmarin D.H., Van den Brink B. (2024). Introduction of Spiritual Psychotherapy for Inpatient, Residential, and Intensive Treatment (SPIRIT) in The Netherlands: Translation and Adaptation of a Psychotherapy Protocol for Mental Health Care. Religions.

[13] Rosmarin D.H., Pirutinsky S., Schuttenberg E.M., Silveri M.M. (2022). Why is Spiritual Psychotherapy for Inpatient, Residential, and Inpatient Treatment (SPIRIT) more effective when provided by nonreligious clinicians?. Psychotherapy.

[14] Schuttenberg E.M., Johnston A.M., Drury M.J., Sneider J.T., Silveri M.M., Rosmarin D.H. (2022). Effects of sexual orientation on Spiritual Psychotherapy for Inpatient, Residential & Intensive Treatment. Psychiatr. Res. Clin. Pract..

[15] Ojo S., Okoye T.O., Olaniyi S.A., Ofochukwu V.C., Obi M.O., Nwokolo A.S., Okeke-Moffatt C., Iyun O.B., Idemudia E.A., Obodo O.R. (2024). Ensuring Continuity of Care: Effective Strategies for the Post-hospitalization Transition of Psychiatric Patients in a Family Medicine Outpatient Clinic. Cureus.

[16] Mayer G., Zafar A., Hummel S., Landau F., Schultz J.H. (2023). Individualisation, personalisation and person-centeredness in mental healthcare: A scoping review of concepts and linguistic network visualisation. BMJ Ment. Health.

[17] Slade M. (2009). Personal Recovery and Mental Illness: A Guide for Mental Health Professionals.

[18] Flick U. (2018). An Introduction to Qualitative Research.

[19] Elo S., Kyngäs H. (2008). The qualitative content analysis process. J. Adv. Nurs..

[20] Giesen L.A., Meijer E.M., Ten Have W.D., van der Weide M. (2020). Structuring a team-based approach to coding qualitative data. Int. J. Qual. Methods.

[21] Elo S., Kääriäinen M., Kanste O., Pölkki T., Utriainen K., Kyngäs H. (2014). Qualitative content analysis: A focus on trustworthiness. SAGE Open.

[22] Burlingame G.M., Strauss B., Joyce A.S. (2013). Change mechanisms and effectiveness of small group treatments. J. Clin. Psychol..

[23] Ghezelseflou M. (2023). The role of spirituality in coping with chronic illness. J. Personal. Psychosom. Res..

[24] Zinnbauer B.J., Pargament K.I. (2011). Pain, spirituality, and meaning making: What can we learn from the literature?. Religions.

[25] Kopelowicz A., Liberman R.P. (2003). Integrating treatment with rehabilitation for persons with major mental illnesses. Psychiatr. Serv..

[26] van Nieuw Amerongen-Meeuse J.C., Schaap-Jonker H., Schuhmann C., Anbeek C., Braam A.W. (2018). The “religiosity gap” in a clinical setting: Experiences of mental health care consumers and professionals. Ment. Health Relig. Cult..

[27] Yalom I.D., Leszcz M. (2005). The Theory and Practice of Group Psychotherapy.

[28] Stickley T., Hui A. (2012). Recovery-oriented mental health services: A review of the evidence. J. Psychiatr. Ment. Health Nurs..

[29] Bhattacharya S., Pradhan K.B., Bashar M.A., Tripathi S., Thiyagarajan A., Srivastava A., Singh A. (2020). Salutogenesis: A Bona Fide Guide towards Health Preservation. J. Family Med. Prim. Care.

[30] Pargament K.I., Feuille M., Burdzy D. (2011). The Brief RCOPE: Current psychometric status of a short measure of religious coping. Religions.

[31] Leamy M., Bird V.J., Le Boutillier C., Williams J., Slade M. (2011). A conceptual framework for personal recovery in mental health: Systematic review and narrative synthesis. Br. J. Psychiatry.

[32] Exline J.J., Pargament K.I., Grubbs J.B., Yali A.M. (2014). The Religious and Spiritual Struggles Scale: Development and initial validation. Psychol. Relig. Spiritual..

[33] The Lancet Public Health (2024). Mental Health: A Public Health Crisis Unfolding. Lancet Public Health.

